# Risk prediction models for esophageal cancer: A systematic review and critical appraisal

**DOI:** 10.1002/cam4.4226

**Published:** 2021-08-20

**Authors:** He Li, Dianqin Sun, Maomao Cao, Siyi He, Yadi Zheng, Xinyang Yu, Zheng Wu, Lin Lei, Ji Peng, Jiang Li, Ni Li, Wanqing Chen

**Affiliations:** ^1^ Office of Cancer Screening National Cancer Center/ National Clinical Research Center for Cancer/ Cancer Hospital Chinese Academy of Medical Sciences and Peking Union Medical College Beijing China; ^2^ Department of Cancer Prevention and Control Shenzhen Center for Chronic Disease Control Shenzhen China

**Keywords:** esophageal cancer screening, individualized risk assessment, prediction models, systematic review

## Abstract

**Background and aims:**

Esophageal cancer risk prediction models allow for risk‐stratified endoscopic screening. We aimed to assess the quality of these models developed in the general population.

**Methods:**

A systematic search of the PubMed and Embase databases from January 2000 through May 2021 was performed. Studies that developed or validated a model of esophageal cancer in the general population were included. Screening, data extraction, and risk of bias (ROB) assessment by the Prediction model Risk Of Bias Assessment Tool (PROBAST) were performed independently by two reviewers.

**Results:**

Of the 13 models included in the qualitative analysis, 8 were developed for esophageal squamous cell carcinoma (ESCC) and the other 5 were developed for esophageal adenocarcinoma (EAC). Only two models conducted external validation. In the ESCC models, cigarette smoking was included in each model, followed by age, sex, and alcohol consumption. For EAC models, cigarette smoking and body mass index were included in each model, and gastroesophageal reflux disease, uses of acid‐suppressant medicine, and nonsteroidal anti‐inflammatory drug were exclusively included. The discriminative performance was reported in all studies, with C statistics ranging from 0.71 to 0.88, whereas only six models reported calibration. For ROB, all the models had a low risk in participant and outcome, but all models showed high risk in analysis, and 60% of models showed a high risk in predictors, which resulted in all models being classified as having overall high ROB. For model applicability, about 60% of these models had an overall low risk, with 30% of models of high risk and 10% of models of unclear risk, concerning the assessment of participants, predictors, and outcomes.

**Conclusions:**

Most current risk prediction models of esophageal cancer have a high ROB. Prediction models need further improvement in their quality and applicability to benefit esophageal cancer screening.

## Graphical abstract

Risk prediction models of esophageal squamous cell carcinoma (ESCC) and esophageal adenocarcinoma (EAC) varied widely across populations, among which more ESCC models were developed in the Asian population, and more EAC models were developed in the Western population. The studies’ quality was not high, mainly due to limitations in the statistical analysis for risk of bias (ROB) and predictors’ availability for applicability.

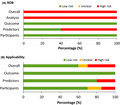

## 1 INTRODUCTION

Esophageal cancer is associated with a heavy disease burden globally. Approximately 0.60 million new cases and 0.54 million deaths related to esophageal cancer were estimated to occur worldwide in 2020, with esophageal cancer ranking eighth in cancer incidence and sixth in cancer mortality.^1^ As its primary histologic subtypes, esophageal squamous cell carcinoma (ESCC) and esophageal adenocarcinoma (EAC) exhibit different geographical distributions, risk factors, and molecular profiles.^2^, ^3^, ^4^ Esophageal cancer, including any histologic subtype, is characterized by a poor prognosis, with a 5‐year survival rate of 10%–30% in most countries.^5^ However, the 5‐year survival rate of patients at an early stage could reach 80% or greater.^6^

Many studies show that endoscopic screening could identify more patients at the early stage and reduce esophageal cancer mortality.^7^, ^8^ Although mass endoscopy screening is urgent for some developing countries with a heavy esophageal cancer burden, it is impractical due to the high cost and limited capability to offer high‐quality endoscopy. Some studies have shown that a risk‐stratified strategy that provides endoscopies to a limited group of individuals with high risk would be preferable to a universal screening strategy.^9^, ^10^, ^11^, ^12^ Risk‐stratified endoscopic screening would improve screening efficiency, avoid unnecessary endoscopies for those assessed as low risk, and reduce screening costs. Prediction models for esophageal cancer are a promising approach to realize risk‐stratified screening by quantifying the individual risk of developing esophageal cancer.^9^, ^10^, ^11^, ^12^

The accuracy and validity of a prediction model play a crucial role in esophageal cancer screening success. An ideal prediction model should perform well in population representation, discrimination, and calibration. In addition, an easy and inexpensive tool is needed for application in the general population.^13^ While there are several prediction models for esophageal cancer, the overall quality is not clear, and which of them could be recommended to guide and inform healthcare providers and payers of their relative merits is uncertain. Therefore, this systematic review aimed to summarize and critically appraise published risk prediction models for esophageal cancer developed in the general population by considering risk of bias (ROB) and population applicability. Each identified model was assessed by the Prediction model Risk Of Bias Assessment Tool (PROBAST).^14^

## 2 METHODS

We performed the systematic review with the protocol published in the International Prospective Register of Systematic Reviews (PROSPERO; registration number: CRD42020202988) and reported this review following the PRISMA (preferred reporting items for systematic reviews and meta‐analyses) statement.

### 2.1 Literature search

The PubMed and Embase databases were systematically searched for English‐language studies published from Jan 1, 2000 to May 31, 2021 reporting on a prediction model for esophageal cancer, including ESCC and EAC. We created the following search algorithm: (predict OR calculate OR assess OR score OR nomogram OR model) AND ((esophageal OR esophagus) AND (cancer OR carcinoma OR adenocarcinoma)) to capture relevant studies, and the details of the research strategy are presented in Table S1. Two researchers (LH, SDQ) performed the literature search independently, and discrepancies were resolved by a third researcher (ZYD). We further manually searched the references of each eligible article for potentially eligible studies.

### 2.2 Eligibility criteria

Table S2 presents the eligibility criteria based on the CHARMS checklist. Briefly, the inclusion criteria were studies that developed or validated a prediction model for esophageal cancer in the general population. The outcome was defined as any pathology of esophageal cancer (ESCC or EAC). The exclusion criteria were as follows: (a) studies performed in animals; (b) studies that did not address the development of a prediction model; (c) studies for which the outcomes included not only esophageal cancer but also precancerous lesions related to esophageal cancer; (d) studies that did not report the area under the curve‐receiver operating characteristic (AUC) and/or the sensitivity and specificity of the prediction model; and (e) articles that were not published in English.

### 2.3 Data extraction and quality assessment

A data extraction form was developed to collect relevant information based on the CHARMS checklist.^15^ For each eligible article, we extracted information on the first author and year of publication, study design, study setting, geographical location, number of participants and number of events, modeling method, number and type of predictors in the final model, definition of the outcome, measures of key predictive performance (discrimination and calibration), and model estimation (internal validation and method and external validation). Potential measures of discrimination mainly included the C statistic and D statistic, and potential measures for the assessment of calibration were the calibration plot, calibration slope, and Hosmer–Lemeshow (H–L) test.^15^

The quality of the studies included in this review was assessed using PROBAST.^14^ This tool has been developed specifically to assess the ROB and applicability for prediction model studies. ROB assessment consisted of 20 signaling questions in four domains of participants, predictors, outcome, and statistical analysis. Applicability assessment consisted of several questions in three domains: participants, predictors, and outcome.

## 3 RESULTS

**FIGURE 1:**
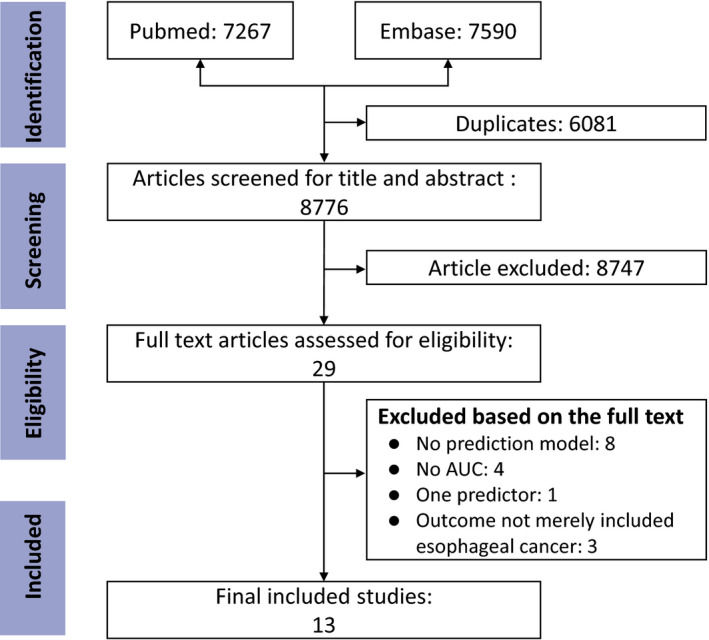
Flow diagram of selection of studies that described the development of a Prediction model for the risk of esophageal cancer based on a systematic literature search

Figure 1 shows the study selection process. We identified 14,857 publications, of which 8776 were not duplicates. After screening titles and abstracts, 29 were retained for the full‐text review. We further excluded 16 publications because they did not address the development of a prediction model (n = 8), did not report the AUC (n = 4), had only one predictor (n = 1), or the outcome included more than esophageal cancer (n = 3). Finally, a total of 13 studies were included.

### 3.1 General characteristics of prediction models

Table 1 summarizes the 13 prediction models’ major characteristics. Among them, nine were diagnostic models^9^, ^10^, ^11^, ^16^, ^17^, ^18^, ^19^, ^20^, ^21^ and the other four were prognostic models.^12^, ^22^, ^23^, ^24^ Eight studies developed prediction models for ESCC, and the other five developed prediction models for EAC. Most models of ESCC (n = 6 [75%]) were developed in Asia (China, Japan, and Iran), and the other two were developed in Europe (Sweden and Norway). The five EAC prediction models were all developed in western counties, including Australia, Sweden, North America, the United Kingdom, and Norway. The sample sizes ranged from 868 ^16^ to 355,034.^12^, ^22^, ^23^, ^24^

**TABLE 1:** Characteristics of model type, design, participant population, and outcome of the included models

First Author (year)	Model Type	Study design	Setting summary	Sex^a^	Country (s)	Sample size		Outcomes	
						Total	Case	Outcome	Outcome details
Yokoyama T (2008)^16^	Diagnostic model	Case‐control	Hospital	M	Japan	868	234	ESCC	Not report
Etemadi A (2012)^17^	Diagnostic model	Case‐control	Community	Both	Iran	871	300	ESCC	Histologically confirmed
Chang J (2013)^18^	Diagnostic model	Case‐control	Community	Both	China	20,298	9805	ESCC	Histologically confirmed
Thrift AP (2013)^9^	Diagnostic model	Case‐control	Community	Both	Australia	1944	364	EAC	Histologically confirmed
Xie SH (2016)^10^	Diagnostic model	Case‐control	Community	Both	Sweden	1009	189	EAC	Histologically confirmed
Dong J (2018)^19^	Diagnostic model	Case‐control	Community mixed with hospital	Both	UK, North America, Western Europe, and Australia	4688	2511	EAC	Histologically confirmed
Kunzmann AT (2018)^12^	Prognostic model	Cohort	Community	Both	UK	355,034	220	EAC	Histologically confirmed
Xie SH (2018)^22^	Prognostic model	Cohort	Community	Both	Norway	62,576	29	EAC	Histologically confirmed
Wang QL (2019)^11^	Diagnostic model	Case‐control	Community	Both	Sweden	987	167	ESCC	Histologically confirmed
Chen W (2021)^23^	Prognostic model	Cohort	Community	Both	China	86,745	298	ESCC	Histologically confirmed
Shen Y (2021)^20^	Diagnostic model	Case‐control	Community mixed with hospital	Both	China	1464	244	ESCC	Histologically confirmed
Yang X (2021)^21^	Diagnostic model	Case‐control	Hospital	M; F	China	3410	1418	ESCC	Histologically confirmed
Wang QL (2021)^24^	Prognostic model	Cohort	Community	Both	Norway	77,476	53	ESCC	Histologically confirmed

Abbreviations: EAC, esophageal adenocarcinoma; ESCC, esophageal squamous cell carcinoma; UK, United Kingdom.

^a^ Models applicable to only men (M), men and women, respectively (M; F), or both sexes (Both).

Most of the models (n = 11 [85%]) were developed by logistic regression, and the other two were developed by competing‐risk regression^22^, ^24^ (Table S3). Missing predictor data existed in twelve models (92%); however, only two studies^12^, ^19^ handled the missing data by imputation procedures. Among the remaining ten models with missing predictors, three models^18^, ^22^, ^23^ excluded missing predictor data from the multivariable regression, and seven models^9^, ^10^, ^11^, ^16^, ^17^, ^21^, ^24^ did not report any techniques to handle missing data (Table S3). All publications employed discrimination methods to assess the prognostic utility of their model with the AUC. However, the steps of evaluating the model performance of calibration were suboptimal. Half of the included studies (n = 7)^9^, ^10^, ^11^, ^16^, ^18^, ^20^, ^21^ did not evaluate model fit through calibration methods. All models except one^21^ were internally validated, and only two^22^, ^24^ were validated by an external population.

### 3.2 Variables in the model

Tables 2 and 3 present the variables included in the prediction models of ESCC and EAC, respectively. Overall, the variables of ten models^9^, ^10^, ^11^, ^12^, ^17^, ^20^, ^21^, ^22^, ^23^, ^24^ were easily obtainable (via medical records or questionnaires), including demographic characteristics, lifestyle risk factors, family history of clinical cancer symptoms, medication use history, disease history, and surgical history. The other three models^16^, ^18^, ^19^ further included genetic factors. The types of included risk factors differed between the ESCC and EAC models. Demographic factors and lifestyle risk factors were frequently used in ESCC models. Cigarette smoking was the most common predictor and was included in all ESCC models, followed by age, sex, and alcohol consumption with a frequency of 75%; education, with a frequency of 50%; predictors of family history of esophageal cancer/upper gastrointestinal cancer and frequency of fruit consumption, with a frequency of 38%; and predictors of frequency of salty food, tea temperature, and body mass index (BMI), with a frequency of 25% (Table 2). For EAC models, cigarette smoking and BMI were the most common predictors and were included in all EAC models. In addition, symptoms of gastroesophageal reflux disease (GERD) and the use of acid‐suppressant medicine, and the use of nonsteroidal anti‐inflammatory drug (NSAID) medication were exclusively included in EAC models, with a frequency of 40% (Table 3).

**TABLE 2:** Predictors included in the eight ESCC prediction models

Predictors	Yokoyama T (2008)^16^	Etemadi A (2012)^17^	Chang, (2013)^18^	Wang QL (2019)^11^	Chen W (2021)^23^	Shen Y (2021)^20^	Yang X (2021)^21^	Wang QL (2021)^24^
Demographic and social economic status								
Age		•	•	•	•	•		•
Sex			•	•	•	•	•	•
Ethnicity		•						
Education		•		•		•	•	
Marital status		•						
Living with a partner/place of residents during childhood				•				
Family wealth score							•	
Lifestyle								
Cigarette smoking	•	•	•	•	•	•	•	•
Alcohol consumption	•		•	•		•	•	•
Frequency of green‐yellow vegetables	•							
Frequency of fruits	•				•	•		
Frequency of salty food					•	•		
Frequency of hot food						•		
Opium use		•						
Tea temperature		•					•	
Water source		•						
Oral hygiene								
Oral health/Frequency of brushing tooth		•					•	
Missing and filled teeth number							•	
Disease history and symptoms								
Disease history of esophagitis or peptic ulcer					•			
Retrosternal pain, back pain, or neck pain					•			
Physical examination								
BMI/Adult height							•	•
Family history								
Esophageal cancer/Upper gastrointestinal cancer		•			•		•	
Genetics								
SNPs			•					
ALDH2 with alcohol consumption	•							
SNPs with alcohol consumption			•					

Abbreviations: ALDH2, aldehyde dehydrogenase‐2; BMI, body mass index; ESCC, esophageal squamous cell carcinoma; SNPs, single nucleotide polymorphisms.

• indicated predictors in the ESCC prediction models.

**TABLE 3:** Predictors included in the five EAC prediction models

Predictors	Thrift AP (2013)^9^	Xie SH (2016)^10^	Dong J (2018)^19^	Kunzmann AT (2018)^12^	Xie SH (2018)^22^
Demographic and social economic status					
Age			•	•	•
Sex			•	•	•
Education	•				
Living with a partner during childhood		•			
Lifestyle					
Cigarette smoking	•	•	•	•	•
Medicine use and symptoms					
Use of NSAID	•		•		
GERD symptoms and/or use of acid‐suppressant medications	•	•			
GERD symptoms			•		•
Physical examination					
BMI	•	•	•	•	•
Disease history					
Esophagitis		•			
Diaphragmatic hernia		•			
Esophageal conditions^a^				•	
Surgeon history					
Gastric or duodenal ulcer		•			
Esophagitis, diaphragmatic hernia, or severe reflux		•			
Genetics					
SNPs			•		

Abbreviations: BMI, body mass index; EAC, esophageal adenocarcinoma; GERD, gastroesophageal reflux disease; NSAID, nonsteroidal anti‐inflammatory drug; SNPs, single nucleotide polymorphisms.

• indicated predictors in the EAC prediction models.

^a^ Esophageal conditions included self‐reported history of gastroesophageal reflux disease, Barrett's esophagus, hiatus hernia, or esophageal stricture and/or esophageal fundoplication or hiatus hernia surgery and/or anti‐reflux medication use (none or any).

### 3.3 Model performance

Figure S1 shows the performance metrics for each model. Of the 13 models, 11 reported C statistics (i.e., AUC) in the derivation cohorts, ranging from 0.76 (95% confidence interval (CI): 0.73–0.79)^9^ to 0.88 (95% CI: 0.83–0.93)^22^ for EAC and from 0.71 (95% CI: 0.70–0.72)^18^ to 0.81 (95% CI: 0.78–0.83)^23^ for ESCC. The other two models^16^, ^17^ did not report the AUC in the derivation cohort but reported it in the internal validation. Six models reported the performance of calibration in the form of a curve (n = 2)^22^, ^24^ or H‐L test (n = 2)^17^, ^19^ or both of these methods (n = 2).^12^, ^23^ Except for one model,^21^ the other eleven models were internally validated. AUCs were slightly lower than those in the derivation cohort, except in the two models^16^, ^17^ that did not report the AUC in the derivation cohort. Only two models were externally validated, with C statistics of 0.89 (95% CI: 0.84–0.94)^22^ and 0.70 (95% CI: 0.64–0.75).^24^

### 3.4 Study quality assessment

**FIGURE 2:**
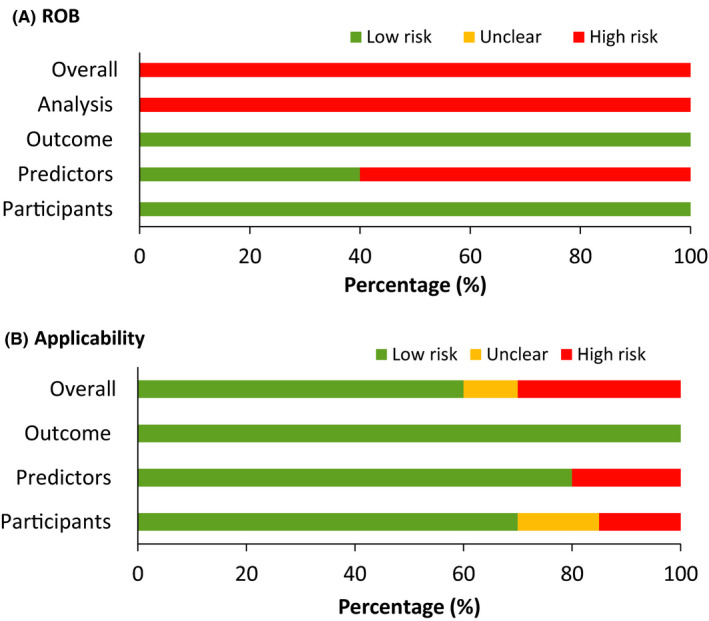
Risk of bias assessment (A) and applicability (B) according to the Prediction model Risk Of Bias Assessment. ROB, risk of bias

Table 4 summarizes the quality assessment results for all included studies, and full details are provided in Figure S2. All the models had a low ROB in the dominance of participants and outcome. In contrast, about 60% of them showed a high ROB in the dominance of predictors as predictor assessments made with knowledge of the outcome, which resulted from case‐control study designs. The analysis criterion also showed a high ROB in all studies because of omitting missing data instead of performing imputation methods (n = 3)^18^, ^22^, ^23^ or without any handling for missing data (n = 7)^9^, ^10^, ^11^, ^16^, ^17^, ^21^, ^24^ and inappropriate performance measures (n = 9).^9^, ^10^, ^11^, ^16^, ^17^, ^18^, ^19^, ^20^, ^21^ By applying PROBAST, all models were classified as having overall high ROB (Figure 2A).

**TABLE 4:** Quality assessment for ROB and applicability concern of the included models

First Author (year)	ROB				Applicability			Overall	
	Participants	Predictors	Outcome	Analysis	Participants	Predictors	Outcome	ROB	Applicability
Yokoyama T (2008)^16^	+	‐	+	‐	‐	‐	+	‐	‐
Etemadi A (2012)^17^	+	‐	+	‐	+	+	+	‐	+
Chang J (2013)^18^	+	‐	+	‐	+	‐	+	‐	‐
Thrift AP (2013)^9^	+	‐	+	‐	+	+	+	‐	+
Xie SH (2016)^10^	+	‐	+	‐	+	+	+	‐	+
Dong J (2018)^19^	+	‐	+	‐	?	‐	+	‐	‐
Kunzmann AT (2018)^12^	+	+	+	‐	+	+	+	‐	+
Xie SH (2018)^22^	+	+	+	‐	+	+	+	‐	+
Wang QL (2019)^11^	+	‐	+	‐	+	+	+	‐	+
Chen W (2021)^23^	+	+	+	‐	+	+	+	‐	+
Shen Y (2021)^20^	+	+	+	‐	?	+	+	‐	?
Yang X (2021)^21^	+	‐	+	‐	‐	+	+	‐	‐
Wang QL (2021)^24^	+	+	+	‐	+	+	+	‐	+

Abbreviation: ROB, risk of bias.

+ indicates low ROB/low concern regarding applicability;

‐ indicates high ROB/high concern regarding applicability;

? indicates unclear ROB/unclear concern regarding applicability.

When the 13 models were assessed according to applicability concerns, about 60% of models (8/13)^9^, ^10^, ^11^, ^12^, ^17^, ^22^, ^23^, ^24^ were considered to have an overall “low risk” following the assessment of applicability to participants, predictors, and outcomes (Table 4; Figure 2B). Four models were assessed as having an overall “high risk” applicability concern because of having applicability to predictors exclusively (n = 2),^18^, ^19^ participants exclusively (n = 1)^21^ or predictors in combination with participants (n = 1).^16^ The remaining study^20^ was assessed as “unclear” overall because of applicability concern in participants.

## 4 DISCUSSION

This systematic review summarizes the 13 risk prediction models for esophageal cancer published since 2000, which is the first review assessing the ROB of risk prediction models for esophageal cancer. Risk prediction models of ESCC and EAC varied widely across populations, among which more ESCC models were developed in the Asian population, and more EAC models were developed in the Western population, and predictors differed in ESCC and EAC prediction models. Although the discriminative performance was mostly acceptable, calibration metrics were not appropriately reported in every model. There is an urgent need for external validation in representative populations because these existing models are available tools for population‐wide risk assessments. Furthermore, the studies’ quality was not high, mainly due to limitations in the statistical analysis for ROB and predictor availability for applicability.

### 4.1 Explanations of major findings

In this systematic review, we found that 75% of ESCC models were developed in the Asian population, and 60% of EAC models were developed in the Western population. This finding agrees with the distribution of ESCC and EAC across the world, where more than 80% of global ESCC cases occur in Asia, and more than half of global EAC cases occur in Western countries.^3^ The distinct difference in predictors in the ESCC and EAC models could be explained by the difference in risk factors for these two subtypes of esophageal cancer. ESCC prediction models included demographic and lifestyle risk factors, such as age, sex, cigarette smoking, and alcohol consumption. These were the four common predictors in ESCC prediction models, and they are risk factors for ESCC with consistent evidence.^25^ Other risk factors with consistent evidence included vegetable and fruit consumption, and hot food and pickled vegetable (salted food) consumption. Some risk variables that were repeatedly reported were poor oral health and opium use,^25^ which also appeared at least once in these included ESCC prediction models. Compared with the predictors used in ESCC models, GERD symptoms, anti‐reflux therapies, and NSAID use were exclusively used in EAC prediction models. These are also common risk factors for EAC.^26^

Although all models had a low ROB in the dominants of participant and outcome, all models showed high ROB in the analysis (Domain 4), which resulted in all models having high ROB according to the PROBAST. Specifically, there are two severe deficiencies in statistical analysis. The first limitation was the inappropriate handling of missing data. Most studies had this issue, among which no information on how missing data was more common^9^, ^10^, ^11^, ^16^, ^17^, ^21^, ^24^ than the exclusion of participants with missing predictors.^18^, ^22^, ^23^ The second limitation was the lack of performance measures. Ensuring that models properly evaluate both calibration and discrimination is a domain on PROBAST (Domain 4.7).^14^ All models reported discriminative performance, with AUCs ranging from 0.71 to 0.88. However, more than half of the studies (n = 7)^9^, ^10^, ^11^, ^16^, ^18^, ^20^, ^21^ did not report model calibration performance, and another 15% (n = 2)^17^, ^19^ only reported statistical tests of calibration instead of calibration plots and tables, which led to “N” in Domain 4.7 of the PROBAST.

More than 75% of the models (n = 10) used predictors that are routinely obtained in clinical or epidemiological settings, which would increase their applicability to daily practice. It is conceivable that a prediction model's performance would improve with the combination of genetic information and biomarkers. However, prediction models with genetic information or biomarkers were identified as high risk in applicability, according to PROBAST, resulting in three models with high risk in applicability. In addition, we found that the addition of genetic risk factors to risk prediction models for esophageal cancer yielded only modest gains in discriminatory power, ranging from 0.70 (0.69–0.71) to 0.71 (0.70–0.72) in a study by *Chang* et al.^18^ and from 0.75 (0.72–0.77) to 0.75 (0.73–0.78) in a study by *Dong* et al.^19^ A study from the UK biobank^27^ identified that the addition of genetic information for EAC did not improve the discriminative performance of a previous prediction model developed with five predictors routinely obtained in clinical practice. It should be carefully considered and thoroughly debated whether biomarkers and genetic information included in the prediction models of esophageal cancer are suitable and feasible to obtain when applying the model to practical situations.

These limitations are also common for prediction models of other cancers.^28^, ^29^ Many reviews have shown that the quality of reporting in published articles describing the development or validation of multivariable prediction models in medicine is insufficient. As such, the Transparent Reporting of a multivariable prediction model for Individual Prognosis or Diagnosis (TRIPOD) statement^13^ was issued. The TRIPOD statement developed a set of recommendations for the reporting of studies developing, validating, or updating a prediction model to improve the quality of the published prediction model studies published in 2015. Approximately 70% of the included prediction models of esophageal cancer published after 2015 could have avoided the issues mentioned above if TRIPOD recommendations had been followed. This finding suggested a lack of experience in the field and low penetration of this statement across professions and regions.

### 4.2 Challenges and further possible directions for clinical and public practice

Several barriers exist to incorporating the existing prediction models of esophageal cancer into clinical practice. The first refers to the representative population. Although the included prediction models presented a low ROB in terms of the domain of participants, the representativeness of participants is still insufficient for the included prediction models of esophageal cancer. Among the 13 included models, there were only four prognostic models based on a prospective cohort design. The remaining nine diagnostic models were not nested case‐control designs, which resulted in the unavailable bias of predictor assessments made without knowledge of outcome in Domain 2.2. Given that the controls were from a community population, which enhances population representativeness, they were assessed as "PY" in Domain 1.1 of the PROBAST. However, two case–control studies included cases only from hospitals, which resulted in the assessment results of high risk in the dominance of participants for applicability. It should be noted that models developed from representative data resources, such as randomized control trials (RCTs), cohorts, nested case‐control studies, and cross‐sectional studies, are still urgently needed.

The second practical challenge for implementing a prediction model as a prescreening tool in the secondary prevention of esophageal cancer is the selection and validity of predictors. Some natural and practical situations increase the difficulties of obtaining valid and reasonable predictors. Different subtypes of esophageal cancer have different genetic markers and risk factors, which is widely accepted.^25^, ^26^ However, there is still a long way to go in the exploration and validation of biomarkers and genetic information of ESCC and EAC with an accuracy that meets the requirements for clinical use.^30^ The distribution of risk factors for a specific subtype of esophageal cancer in different populations may also affect the applicability of the existing models to different contexts. These differences may affect a model's discriminatory accuracy, that is, they may affect a prediction model's practical value.

The third challenge is that most models of esophageal cancer have not been validated in diverse populations. Among the 13 included esophageal cancer models, only an ESCC model and an EAC model were conducted in an external population and demonstrated good performance, with AUCs of 0.89^22^ and 0.70,^24^ respectively. These two models were developed from the same cohort in Norway, and the EAC model was developed to predict the individual 15‐year risk. The time interval may be too long as a risk prediction tool in a cancer screening program, and individual behaviors are likely to change over such wide time intervals, which may weaken the predictive accuracy. No prediction models for esophageal cancer have been externally validated in Asia, which possesses the hugest disease burden of esophageal cancer around the world. To the best of our knowledge, some external validation studies in different settings and countries or comparing several models in an external population have been conducted for female breast cancer,^31^ lung cancer,^32^ and colorectal cancer.^33^ These studies may help understand the existing models’ performance for a specific context and provide robust evidence for policy‐makers or guidelines to select or set suitable strategies for themselves. More external validation studies should be attempted for esophageal cancer prediction models.

Another urgent challenge is how to define “high‐risk individuals” for esophageal cancer screening guidelines. The selection of thresholds to identify high‐risk individuals is the ultimate aim of a prediction model. Many of the included studies demonstrated that a risk‐stratified strategy for endoscopic screening would be more beneficial than a universal screening strategy. However, it should be noted that none of the existing stratified approaches was externally validated, which significantly limited their application potential of discriminating high‐risk individuals from the general population. In addition, the recommendations for screening for the high‐risk population must be flexible and based on different practical situations instead of a one‐size‐fits‐all approach. These recommendations need to be carefully determined by considering the potential benefits and harms to individuals, health resource utilization, esophageal cancer incidence in the population, and healthcare provider and practitioner perspectives.^22^ However, differences in esophageal cancer subtypes and differences in incidence rates existed among countries and even among different regions within a country, which may present a tough challenge for this field. Many cost‐effectiveness analyses have been conducted to select suitable screening criteria for high‐risk populations for cancers of the lung,^34^, ^35^ breast,^36^, ^37^ and prostate.^38^ There were no corresponding studies for esophageal cancer, and more attempts to perform these studies should be made in the future.

## 5 STRENGTHS AND LIMITATIONS OF THE STUDY

This study's main strength is that it provides a comprehensive mapping of the available research on diagnostic and prognostic models of esophageal cancer in the general population, providing comprehensive and objective evidence for policy‐makers. We used a sound methodological review following international guidelines for systematic reviews of diagnostic and prognosis models to search and present a detailed description of the characteristics of the existing esophageal cancer models. Furthermore, we used PROBAST, a new quality assessment tool for risk prediction models, to perform a robust assessment of the ROB for each risk model to understand the overall quality of the current prediction models of esophageal cancer.

The main limitation of this study is that we only included studies published in English and did not systematically search gray literature. Therefore, some models may not be identified. Three prediction models developed in the Chinese population with the outcomes of precancerous lesions and ESCC were excluded because these studies did not report the outcome of ESCC separately from other outcomes, which was not suitable for this study's scope. We acknowledge that further assessment could compare prediction models for precancerous lesions with those for esophageal cancer, as they are both positive cases for endoscopic screening.

## 6 CONCLUSIONS

In this systematic review, we identified and assessed 13 esophageal cancer prediction models. The models present substantial heterogeneity concerning the study population, including risk factors, the statistical methodology of model development, and predicted outcomes. The existing esophageal cancer risk prediction models have a relatively high ROB, with the leading limitation of lacking a standardized and complete statistical methodology for model development and an extreme lack of external validation. Participants and predictors in the current prediction models were two major dominants to restrict the applicability and generalizability. Prediction models need further improvement in their quality and usability to benefit esophageal cancer screening.

## CONFLICTS OF INTEREST

The authors declare no potential conflicts of interest.

## AUTHORS’ CONTRIBUTIONS

H L, DQ S, MM C, SY H, J L, N L, L L, J P, and WQ C contributed to the study concept and design. H L and DQ S contributed to the screening, data abstraction. H L, DQ S, and YD Z contributed to quality assessment of included studies. HL and DQ S collaborated in drafting the manuscript and revising it critically for important intellectual content. All authors contributed to the data interpretation and approved the final manuscript.

## ETHICS APPROVAL AND CONSENT TO PARTICIPATE

Neither specific patient consent nor ethics committee's approval were required because we used published articles that were obtained from open access databases.

## Supporting information

Supplementary Material

## Data Availability

The datasets analyzed during the current study are publicly available from the corresponding author.
