# Supplementary material for: Risk prediction models for esophageal cancer: A systematic review and critical appraisal
Source: Cancer Med. 2021 Aug 20;10(20):7265–76. doi: 10.1002/cam4.4226 (PMC8525074; doi:10.1002/cam4.4226)
Supplement: Supplementary file 1 — Supplementary Material [file CAM4-10-7265-s001.docx]

**Supplementary Table 1.** Search strategy in PubMed and Embase

| Search strategy in PubMed | |
| --- | --- |
| #1 | predict[tiab] OR calculat[tiab] OR assess[tiab] OR scor[tiab] OR nomogram[tiab] OR model[tiab] |
| #2 | esophageal[tiab] OR esophagus[tiab] |
| #3 | cancer[tiab] OR carcinoma[tiab] OR adenocarcinoma[tiab] |
| #4 | esophageal cancer[MeSH Terms] |
| #5 | #2 AND #3 |
| #6 | #4 OR #5 |
| #7 | English[Language] |
| #8 | Journal Article[Publication Type] |
| #9 | 2000/01/01:2021/5/31[Date - Publication] |
| #10 | #1 AND #6 AND #7 AND #8 AND #9 |
| Search strategy in Embase | |
| #1 | predict:ab, ti OR calculat:ab, ti OR assess:ab, ti OR scor:ab, ti OR nomogram:ab, ti OR model:ab, ti |
| #2 | esophageal:ab, ti OR esophagus:ab, ti |
| #3 | cancer:ab, ti OR carcinoma:ab, ti OR adenocarcinoma:ab, ti |
| #4 | 'esophagus cancer'/exp |
| #5 | #2 AND #3 |
| #6 | #4 OR #5 |
| #7 | english:la |
| #8 | article:it AND [2000-2021]/py |
| #9 | #1 AND #6 AND #7 AND #8 |

**Supplementary Table 2.** Criteria to guide the literature search and selection criteria

| **Concept** | **Criteria** |
| --- | --- |
| 1. Prognostic or diagnostic prediction model? | Both, this review focuses on diagnostic and prognostic risk scores for esophageal cancer. |
| 2. Intended scope of the review | Diagnostic/prognostic models to inform physicians, researchers and general population about their current risk (i.e., diagnostic) or the further risk of esophageal cancer (i.e., prognostic). |
| 3. Type of prediction modeling studies | Focus on three types: (i) diagnostic/prognostic models with external validation; and (ii) diagnostic/prognostic models without external validation; (iii) diagnostic/prognostic models of validation. |
| 4. Target population to whom the prediction model applies | General adult population. |
| 5. Outcome to be predicted | Esophageal cancer (both histopathological types of esophageal squamous cell carcinoma and esophageal adenocarcinoma). |
| 6. Time span of prediction | Any. |
| 7. Intended moment of using the model | Prediction models to be used in general adults to ascertain current status of esophageal cancer (i.e., diagnostic) or future risk of esophageal cancer (i.e., prognostic); these models could be used for research purposes, screening in primary and secondary prevention. |

**Supplementary table 3.** Summary of methods of the included models

| **Study (First Author, year)** | **Model Development** | | | |  | **Model Evaluation** | | |
| --- | --- | --- | --- | --- | --- | --- | --- | --- |
|  | **Selection of candidate predictors** | **Regression method** | **Miss data of predictors** | **Methods for handling missing data for model development** |  | **Method of discrimination assessed** | **Method of calibration assessed** | **Method of Internal validation assessed** |
| Yokoyama T (2008) ^16^ | Risk factors drawn from a previous study, then a multivariable logistic regression was used. | Logistic | Yes | Not report |  | AUC | Not report | Cross-validation |
| Etemadi A  (2012) ^17^ | Risk factors drawn from previous studies, then a multivariable logistic regression was used. | Logistic | Yes | Not report |  | AUC | H-L test | Cross-validation |
| Chang J  (2013) ^18^ | SNPs and non-genetic variables were identified in prior studies. | Logistic | Yes | Excluded from the multivariate regression |  | AUC | Not report | Cross-validation |
| Thrift AP  (2013) ^9^ | Predictive variables were identified based on literature, and then were estimated by logistic regression selection. | Logistic | Yes | Not report |  | AUC | Not report | Cross-validation |
| Xie SH  (2016) ^10^ | Predictive variables were identified based on literature, and then were estimated by logistic regression selection. | Logistic | Yes | Not report |  | AUC | Not report | Cross-validation |
| Dong J  (2018) ^19^ | Genetic variants were identified at genome-wide significance in published GWAS. No details given for other variables. All candidate predictors were estimated by logistic regression selection. | Logistic | Yes | Post-imputation |  | AUC | H-L test | Cross-validation; Bootstrap |
| Kunzmann AT (2018) ^12^ | Predictive variables were identified based on literature, and then were estimated by logistic regression selection. | Logistic | Yes | Multiple imputation |  | AUC | H-L test and calibration curve | Bootstrap |

**Supplementary table 3.** Summary of methods of the included models (continuous)

| **Study (First Author, year)** | **Model Development** | | | |  | **Model Evaluation** | | |
| --- | --- | --- | --- | --- | --- | --- | --- | --- |
|  | **Selection of candidate predictors** | **Regression method** | **Miss data of predictors** | **Methods for handling missing data for model development** |  | **Method of discrimination assessed** | **Method of calibration assessed** | **Method of Internal validation assessed** |
| Xie SH  (2018) ^22^ | Predictive variables were identified based on literature, and then were estimated by competing-risk regression. | competing-risk regression | Yes | Excluded from the multivariate regression |  | AUC | Calibration curve | Cross-validation |
| Wang QL  (2019) ^11^ | Predictive variables were identified based on literature, and then were estimated by logistic regression selection. | Logistic | Yes | Not report |  | AUC | Not report | Cross-validation |
| Chen W  (2021) ^23^ | Predictive variables were identified based on literature, and then were estimated by logistic regression selection. | Logistic | Yes | Excluded from the multivariate regression |  | AUC | H-L test and calibration curve | Cross-validation |
| Shen Y  (2021) ^20^ | Predictive variables were identified based on literature, and then were estimated by logistic regression selection. | Logistic | No | Not relative |  | AUC | Not report | Cross-validation |
| Yang X  (2021) ^21^ | Predictive variables were identified based on logistic model and Nomogram model. | Logistic | Yes | Not report |  | AUC | Not report | Not report |
| Wang QL  (2021) ^24^ | Predictive variables were identified based on a previous study and literature, and then were estimated by competing-risk regression. | competing-risk regression | Yes | Not report |  | AUC | Calibration curve | Cross-validation |

Abbreviation: H-L, Hosmer-Lemeshow.

**
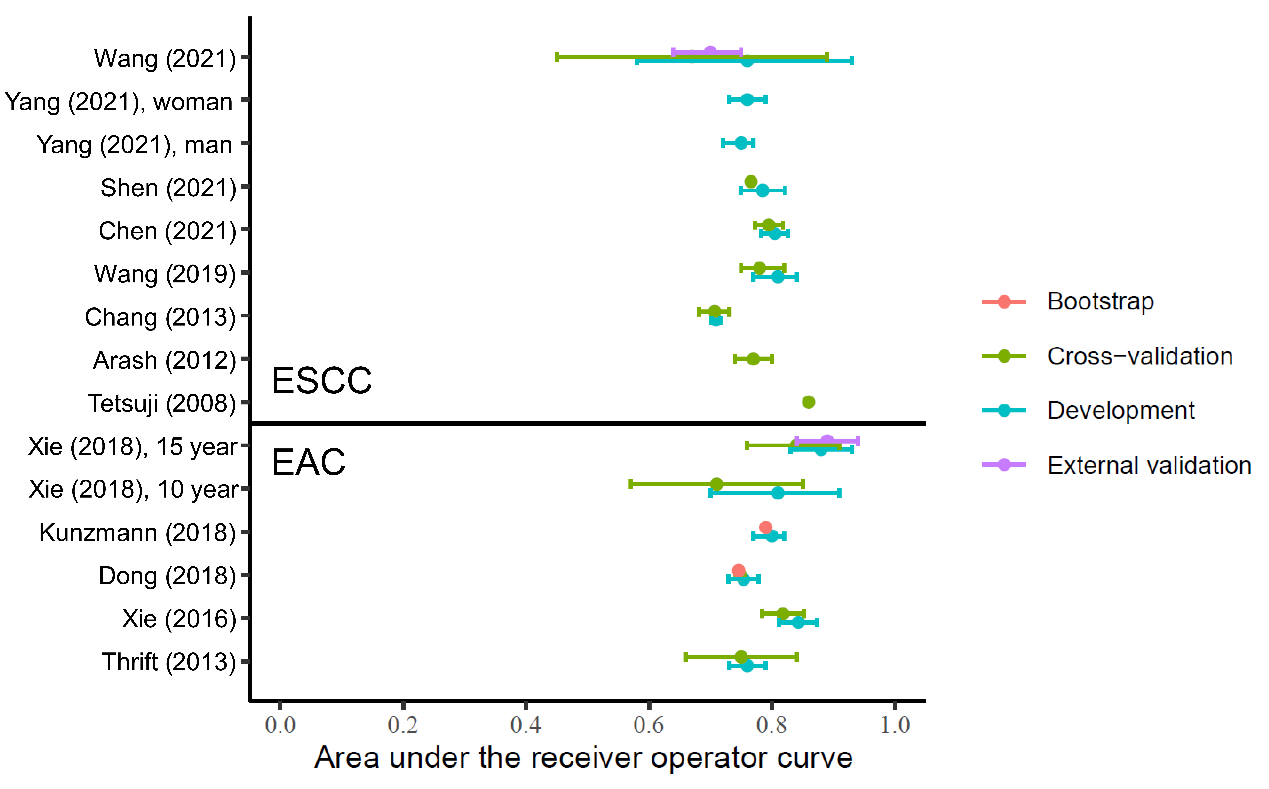
**

**Supplementary Figure 1.** Discriminative performance of the included models.

Abbreviations: ESCC, esophageal squamous cell carcinoma; EAC, esophageal adenocarcinoma.


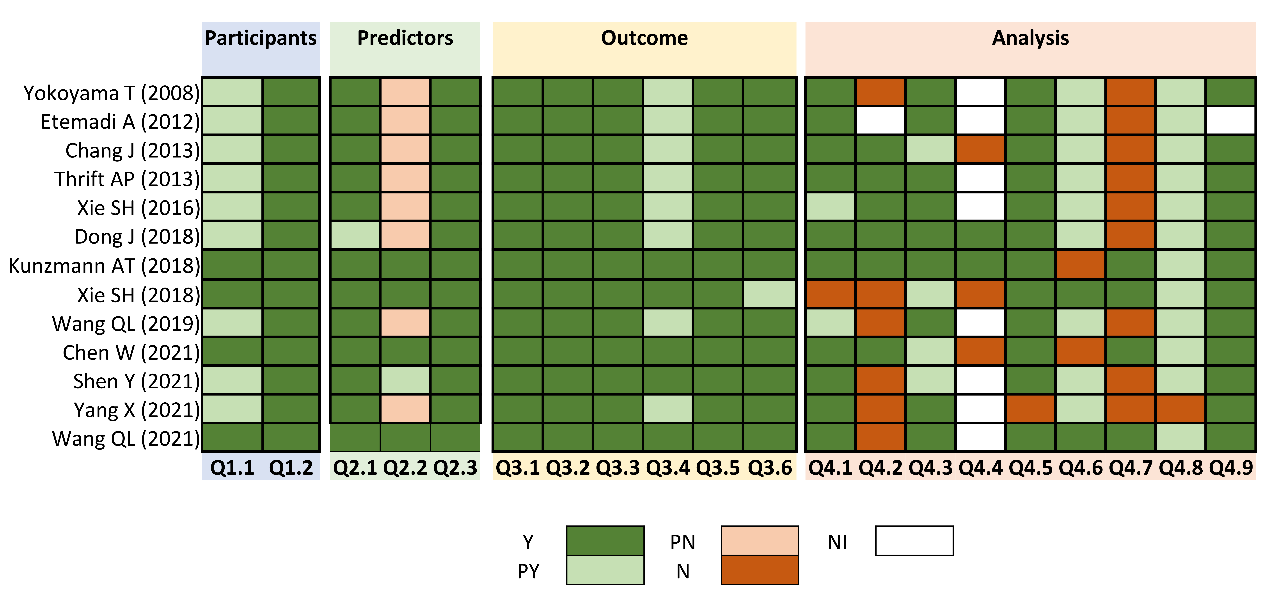


**Supplementary Figure 2.** The quality assessment of the included models. Each signal question was answered as yes (Y, denoted by dark green), probably yes (PY, denoted by light green), no (N, indicated by dark red), probably no (PN, indicated by light red), or no information (NI, denoted by white). Q = question.

**Q1.1:** Were appropriate data sources used, e.g., cohort, RCT, or nested case–control study data?

**Q1.2:** Were all inclusions and exclusions of participants appropriate?

**Q2.1:** Were predictors defined and assessed in a similar way for all participants?

**Q2.2:** Were predictor assessments made without knowledge of outcome data?

**Q2.3:** Are all predictors available at the time the model is intended to be used?

**Q3.1:** Was the outcome determined appropriately?

**Q3.2:** Was a prespecified or standard outcome definition used?

**Q3.3:** Were predictors excluded from the outcome definition?

**Q3.4:** Was the outcome defined and determined in a similar way for all participants?

**Q3.5:** Was the outcome determined without knowledge of predictor information?

**Q3.6:** Was the time interval between predictor assessment and outcome determination appropriate?

**Q4.1:** Were there a reasonable number of participants with the outcome?

**Q4.2:** Were continuous and categorical predictors handled appropriately?

**Q4.3:** Were all enrolled participants included in the analysis?

**Q4.4:** Were participants with missing data handled appropriately?

**Q4.5:** Was selection of predictors based on univariable analysis avoided? [development studies only]

**Q4.6:** Were complexities in the data (e.g., censoring, competing risks, sampling of control participants) accounted for appropriately?

**Q4.7:** Were relevant model performance measures evaluated appropriately?

**Q4.8:** Were model overfitting and optimism in model performance accounted for? [development studies only]

**Q4.9:** Do predictors and their assigned weights in the final model correspond to the results from the reported multivariable analysis? [development studies only]
